# Maternal diabetes causes abnormal dynamic changes of endoplasmic reticulum during mouse oocyte maturation and early embryo development

**DOI:** 10.1186/1477-7827-11-31

**Published:** 2013-04-19

**Authors:** Chun-Hui Zhang, Wei-Ping Qian, Shu-Tao Qi, Zhao-Jia Ge, Ling-Jiang Min, Xiu-Lang Zhu, Xin Huang, Jing-Ping Liu, Ying-Chun Ouyang, Yi Hou, Heide Schatten, Qing-Yuan Sun

**Affiliations:** 1 Department of Reproductive Medicine, Peking University Shenzhen Hospital, Medical Center of Peking University, Shenzhen, Guangdong, China; 2 State Key Laboratory of Reproductive Biology, Institute of Zoology, Chinese Academy of Sciences, Beijing, China; 3 Department of Veterinary Pathobiology, University of Missouri, Columbia, MO, 65211, USA; 4 College of Animal Science and Technology, Qingdao Agricultural University, Qingdao, 266109, China

**Keywords:** Endoplasmic reticulum, Maternal diabetes, Oocyte maturation, Early embryo

## Abstract

**Background:**

The adverse effects of maternal diabetes on oocyte maturation and embryo development have been reported.

**Methods:**

In this study, we used time-lapse live cell imaging confocal microscopy to investigate the dynamic changes of ER and the effects of diabetes on the ER’s structural dynamics during oocyte maturation, fertilization and early embryo development.

**Results:**

We report that the ER first became remodeled into a dense ring around the developing MI spindle, and then surrounded the spindle during migration to the cortex. ER reorganization during mouse early embryo development was characterized by striking localization around the pronuclei in the equatorial section, in addition to larger areas of fluorescence deeper within the cytoplasm. In contrast, in diabetic mice, the ER displayed a significantly higher percentage of homogeneous distribution patterns throughout the entire ooplasm during oocyte maturation and early embryo development. In addition, a higher frequency of large ER aggregations was detected in GV oocytes and two cell embryos from diabetic mice.

**Conclusions:**

These results suggest that the diabetic condition adversely affects the ER distribution pattern during mouse oocyte maturation and early embryo development.

## Background

Women with poorly controlled type I diabetes encounter a higher prevalence of reproductive problems, such as infertility, miscarriage and offspring with congenital malformations [1]. Earlier studies showed that the diabetic condition adversely affects the development of pre- and post-implantation embryos in rodents [2-5]. Furthermore, numerous reports have suggested that *in vitro*-cultured two-cell stage embryos that were recovered from diabetic mice still experience significant delay in their progression to the blastocyst stage and about 50% of two-cell stage embryos isolated from sub-diabetic rats were unable to develop to the eight-cell stage, even in a non diabetic tract [4,6]. Other studies revealed that preovulatory oocytes from chemically induced diabetic mice experience delayed germinal vesicle (GV) breakdown and abnormal cellular metabolism [4,7-9]. Oocytes from diabetic mice displayed a higher frequency of spindle defects and chromosome misalignment in meiosis, resulting in increased aneuploidy rates in ovulated oocytes [10]. To date, however, the effects of maternal diabetes on cytoplasmic structures of oocytes and early embryos remain poorly understood.

During maturation, the oocyte undergoes numerous cytoplasmic changes in preparation for successful fertilization and early embryonic development. One of these changes involves acquiring the ability of the mature oocyte to release Ca^2+^ which is critical for preventing polyspermy and stimulating the oocyte to complete meiosis and begin early development [11]. An important component of the Ca^2+^ release system is the endoplasmic reticulum (ER), which is a multifunctional organelle that consists of a network of membraneous tubules extending throughout the cell [12]. In maturing oocytes, the ER undergoes distribution changes that are associated with the ability of the oocyte to be fertilized successfully [13-20]. ER reorganization is characterized by the development of cortical clusters of ER; this formation correlates with the ability of the maturing oocyte to generate Ca^2+^ transients in response to sperm and inositol 1,4,5-trisphosphate (InsP3) [19]. The presence of cortical ER clusters in mammalian oocytes has been proposed to account for the susceptibility of the cortex to sperm factors and InsP3 [21] and the spatial organization of the sperm-induced Ca^2+^ wave [22]. The similar distribution of the ER clusters and InsP3 receptors (Insp3Rs) further suggests that the ER clusters are specialized sites for the initiation and propagation of Ca^2+^ waves in oocytes [15,17,20]. Changes in the ER distribution patterns also take place after fertilization [12,19]. The spindle-associated ER is seen in most mitotic cells, including those in early stage embryos and in somatic cells during development [12,20].

The objective of this study was to examine how diabetes affects oocyte and embryo quality in relation to the ER distribution pattern as a cytoplasmic criterion. By employing time-lapse live cell imaging confocal microscopy, we revealed dynamic changes of ER structure and found that the diabetic condition adversely affects the distribution pattern of ER during mouse oocyte maturation, fertilization and early embryo development.

## Methods

### Chemicals

All chemicals and media were purchased from Sigma Chemical Company (St. Louis, MO) unless stated otherwise.

### Preparation of mice

Male (3–4 months old) and female (6–8 weeks old) ICR mice (Vital River, Beijing) were used in all experiments. All mouse care and use protocols were employed in accordance with the Animal Research Committee guidelines of the Institute of Zoology (IOZ), Chinese Academy of Sciences. To generate the diabetic mouse model, female ICR mice (age 6-8 weeks) received a single injection of streptozotocin (STZ) at a dose of 190 mg/kg. Four days of injection, a tail-blood sample was measured for glucose concentrations via a Hemocue B glucose analyzer (Stockholm, Sweden). If glucose levels were greater than 300 mg/dl, the animal was selected for use as a diabetic model. Females without injection of STZ served as control. The number of mice used for each experiment is indicated in the figure legends or tables.

### Oocyte and embryo collection and culture

**Figure 1:**
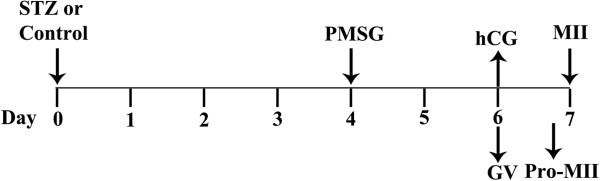
**Schematic illustration of diabetes induction, insulin treatment, and oocyte collection.** Female mice received an injection of 190 mg/kg STZ on d 0. Four days later, blood glucose levels were determined via a commercial glucometer. Mice exhibiting blood glucose levels of at least 300 mg/dl were considered to be diabetic and then were administrated with 10 IU PMSG (d 4). Two days after PMSG, the ovaries were isolated, and cumulus-enclosed GV oocytes were collected (d 6). To retrieve Pro-MII and ovulated oocytes, mice were injected with 10 IU hCG 2 d after PMSG. At 8 h and 13.5 h of hCG administration, oocytes were collected from ovaries and oviductal ampullae (d 7), respectively.

To collect fully grown GV oocytes (Figure 1), control and diabetic mice were injected with 10 IU pregnant mares serum gonadotropin (PMSG) by intraperitoneal injection, and 48 h later, cumulus-enclosed oocytes were obtained by manual rupturing of antral ovarian follicles. To collect Pro-MI and ovulated oocytes, control and diabetic mice received an injection of 10 IU human chorionic gonadotropin (hCG) 2 d of PMSG priming. Oocytes were recovered from the ovary at 8 h and from the oviductal ampullae at 13.5 h of hCG, and cumulus cells were removed by brief incubation in 1 mg/ml hyaluronidase. To collect embryos *in vivo*, estrous females were mated to the males; one-cell (pronuclear stage) and two-cell stage embryos were collected from hormone-injected mice at 27-28 h and 48 h post-hCG, respectively.

Embryos were cultured in KSOM/AA medium containing 0.2 mmol/l glucose, 0.2 mmol/l pyruvate and 10 mmol/l lactate. Such KSOM/AA medium supports development from fertilization to the two-cell stage [23]. For *in vitro* embryo culture, one-cell stage embryos were used for culture after 5 times washing in KSOM/AA medium [23] used for subsequent culture. Finally, they were transferred in groups of 15–30 embryos to pre-equilibrated (5% CO_2_ in 95% air, 37°C) 60 μl drops of KSOM/AA medium under mineral oil (Dow Corning, UK) and placed in a water-jacketed 37°C incubator (Kendro, UK). All embryos were cultured for 2 days until the desired stage of development.

### Imaging of ER

**Figure 2:**
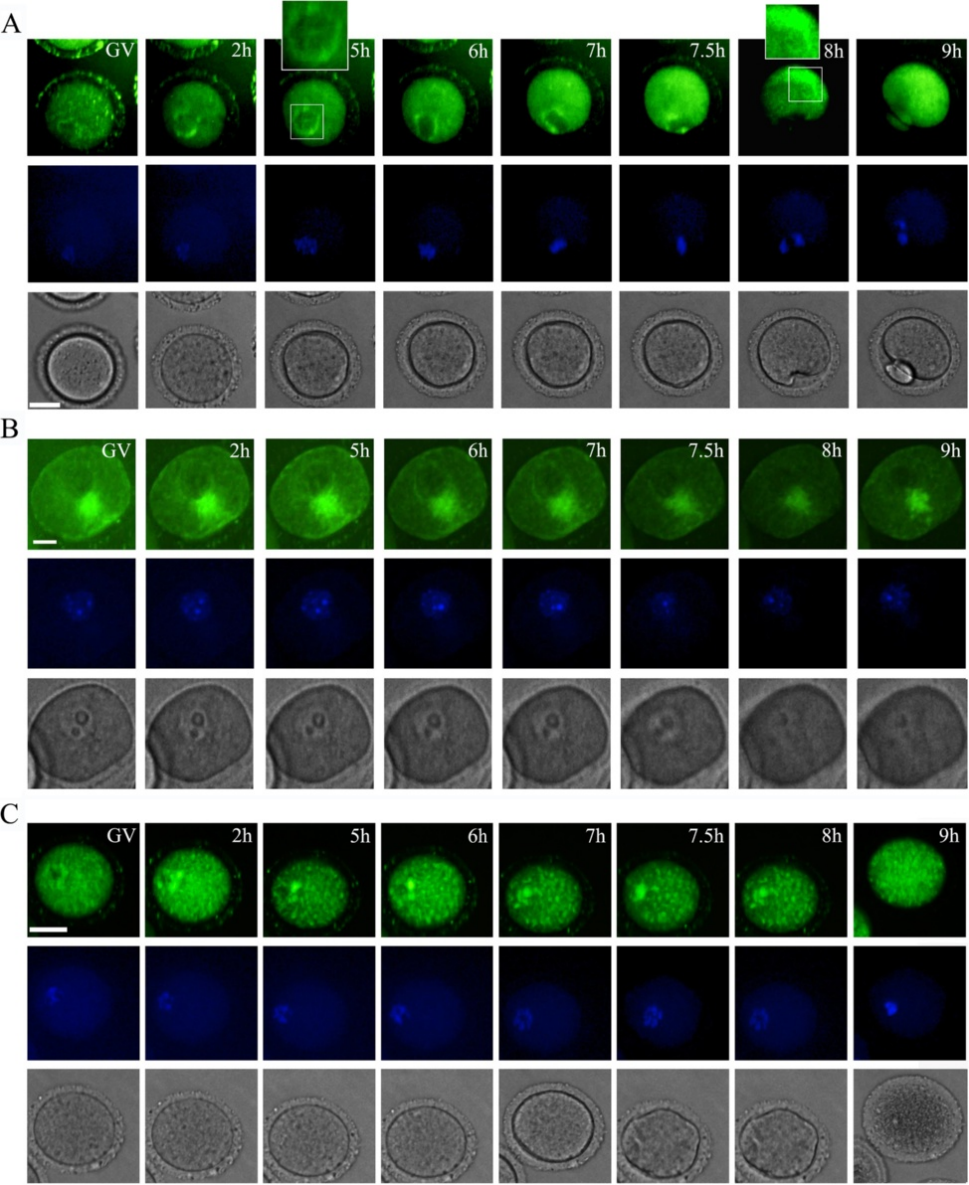
**Maternal diabetes disrupts endoplasmic reticulum redistribution during oocyte maturation *in vitro.*** Time-lapse phase contrast microscopy (bottom row), ER tracker-stained ER (top row) and Hoechst-stained chromosomes (middle row) of a representative control mouse oocyte (from a total of 15 pooled from 1 control mouse, **A**) or diabetic mouse oocyte (from a total of 32 pooled from 3 control mice, **B** and **C**). Times after GVBD (Germinal Vesicle Breakdown) are indicated in the upper right corner. The scale bars represent 10 μm.

**Figure 3:**
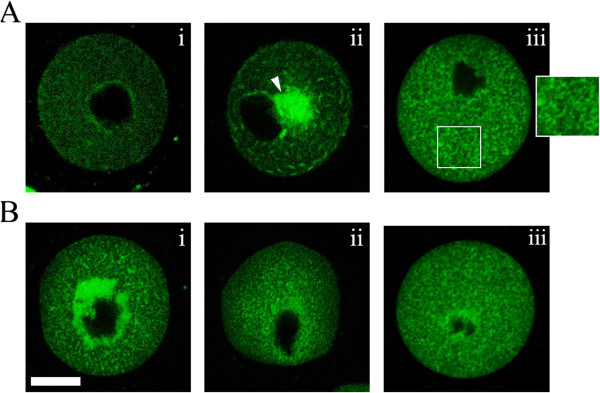
**Maternal diabetes induces ER redistribution defects during *in vivo *oocyte maturation (GV and Pro-MII stages).** GV and Pro-MII oocytes collected from control and diabetic mice were labeled with ER Tracker Green to visualize ER localization. ER distribution patterns were evaluated using fluorescence microscopy. **A**, in fully grown GV oocytes, three distinct patterns of ER distribution were detected: homogeneous distribution pattern (i), ER clouds (white arrows) (ii), and cluster distribution patterns (iii); **B**, in Pro-MII oocytes, three different ER distribution patterns were identified: perinuclear distribution pattern (i), homogeneous distribution (ii), and clustering distribution (iii). Bar = 20 μm.

**Figure 4:**
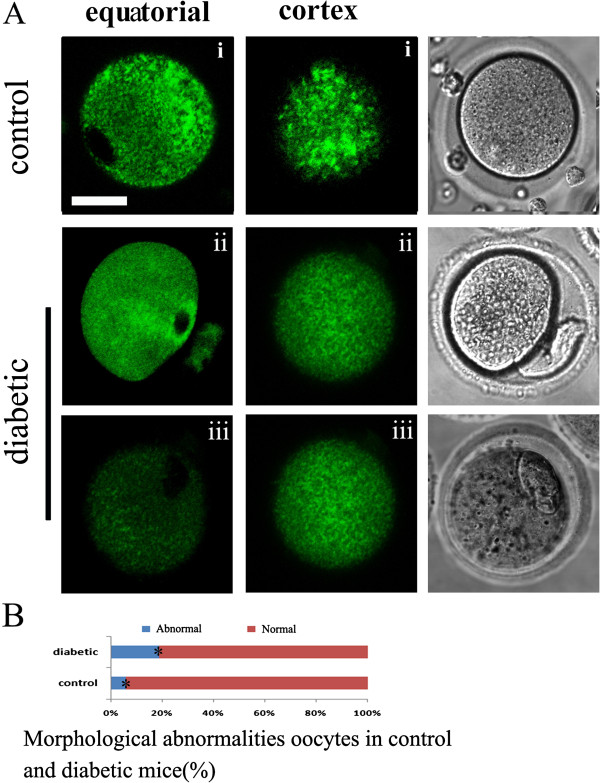
**Maternal diabetes induces ER redistribution defects in MII oocytes.** MII oocytes were labeled with ER Tracker Green to visualize ER localization. ER distribution patterns were evaluated using fluorescence microscopy. Equatorial (left) and cortical (middle) sections are shown. **A**, in MII oocytes, three distinct patterns of ER distribution in equatorial sections and two distribution patterns in cortical sections were detected: no labeling in the area assumed to be the meiotic spindle poles (left i), perinuclear distribution pattern (left ii), and small areas of ER fluorescence within the deeper cytoplasm (left iii); cortical cluster distribution pattern (middle i) and absence of cortical clusters (middle ii). **B**, quantitative analysis of oocytes with morphological abnormalities in control and diabetic mice. The numbers of oocytes scored were 45 pooled from 3 control mice and 39 pooled from 4 diabetic mice. Bar = 20 μm.

ER Tracker Green (glibenclamide BODIPY FL Molecular Probes Eugene, OR) was used as directed by the manufacturer. ER-Tracker™ dyes are cell-permeant live-cell stains that are highly selective for the endoplasmic reticulum (ER). These dyes rarely stain mitochondria, unlike the conventional ER stain DiOC6, and staining at low concentrations does not appear to be toxic to cells. ER Tracker was used at a final concentration of 100-500 mM in KSOM/AA medium. There was no optical interference between the green (ER Tracker) and blue (Hoechst 33342) channels using confocal microscopy. Cells were analyzed at 37°C on a confocal laser-scanning microscope (Zeiss LSM 510 META, Germany). The optical slice (z-dimension) was set to 0.5 μm. Other settings that were used (such as laser intensity and gain value) were adapted to obtain optimal signal to noise ratios. One image was taken from the middle of the stack and prepared for use in Figures 2, 3 and 4 using the Zeiss LSM510 software.

Live oocytes were examined with a confocal laser scanning microscope (Zeiss LSM 510 META, Germany).

### Assessment of ER distribution patterns

Based on previous reports [24], we classified the GV oocytes into three categories: homogeneous distribution pattern (i), ER clouds (ii), and cluster distribution patterns (iii). In Pro-MI oocytes, three different ER distribution patterns were identified: perinuclear distribution pattern (i), homogeneous distribution (ii), and clustering distribution (iii). In MII oocytes, three distinct patterns of ER distribution in equatorial sections and two distribution patterns in cortical sections: no labeling in the area assumed to be the meiotic spindle poles (i), perinuclear distribution pattern (ii), and small areas of ER fluorescence within the deeper cytoplasm (iii); cortical cluster distribution pattern (i) and without cortical clusters (ii). In one-cell stage embryos, two distinct patterns of ER distribution were detected: perinuclear distribution, and larger areas of fluorescence deeper within the cytoplasm (i), homogeneous distribution pattern (ii). In two-cell stage embryos, three different distribution patterns of ER were identified: perinuclear distribution pattern (i), homogeneous distribution (ii), and large aggregated ER (iii).

### Imaging experiments

ER dynamics were recorded on a Perkin Elmer precisely Ultra VIEW VOX confocal Imaging System. We used a narrow band pass EGFP filter set and a 30% cut neutral density filter from Chroma. Exposure time was set ranging between 300 and 800 ms depending on the ER tracker and Hoechst 33342 fluorescence levels. The acquisition of digital time-lapse images was controlled by IP Lab (Scanalytics) or AQM6 (Andor/Kinetic-imaging) software packages. Confocal images of ER in live oocytes were acquired with a 20 × oil objective on a spinning disk confocal microscope (Perkin Elmer). The time-lapse images were acquired every 30 min for 10–12 hr (live oocyte maturation in vitro) or13-15 hr (early embryo development).

Data analysisTests of statistical significance were performed using SPSS software (SPSS Inc, Chicago, IL). Differences between groups were determined by Chi-square test or Fisher exact test. P <0.05 was considered statistically significant.

## Results

### Maternal diabetes disrupts endoplasmic reticulum redistribution during oocyte maturation *in vitro*

To determine whether maternal diabetes influences the redistribution of endoplasmic reticulum during oocyte maturation, we recorded dynamic changes of endoplasmic reticulum during mouse oocyte maturation *in vitro* with time-lapse microscopy. Diabetic mouse oocytes (two representative oocytes of a total of 32 pooled from 3 diabetic mice) displayed significantly higher morphologically abnormal characteristics than controls (Figure 2B, C and see Additional file 1: Supplemental Video S1, Additional file 2: Supplemental Video S2). As shown in Figure 2B and Figure 2C, a homogeneous distribution of ER clusters throughout the entire ooplasm could simultaneously be detected during the entire meiotic maturation process in oocytes from diabetic mice. Importantly, oocytes from diabetic mice displayed a significantly higher percentage of aggregated ER distribution near the nucleus compared to the controls. Moreover, the oocytes displaying aggregated ER at the GV stage were not able to resume meiotic maturation and completely deteriorated within a short time. In contrast, as shown in Figure 2A and Additional file 3: Supplemental Video S3 (a representative control mouse oocyte of a total of 15 pooled from 1 control mouse), the germinal vesicle membrane was prominently labeled by ER tracker. Following GVBD a distinctive ER ring indicated an enriched localization around the nucleus. It was observed in the center of the oocyte and became translocated toward the cortex, followed by formation of ER clusters that resided in the vegetal cortex. It was characterized by the cortical cytoplasm labeled by ER tracker with apparently brighter staining in normal vs diabetic oocytes. Thus, cortical clusters of ER were formed in the later stages of oocyte maturation, close to the time of the first polar body (Pb1) extrusion (8 h of GVBD, Figure 2A).

### Maternal diabetes induces ER redistribution defects during *in vivo* oocyte maturation

**Figure 5:**
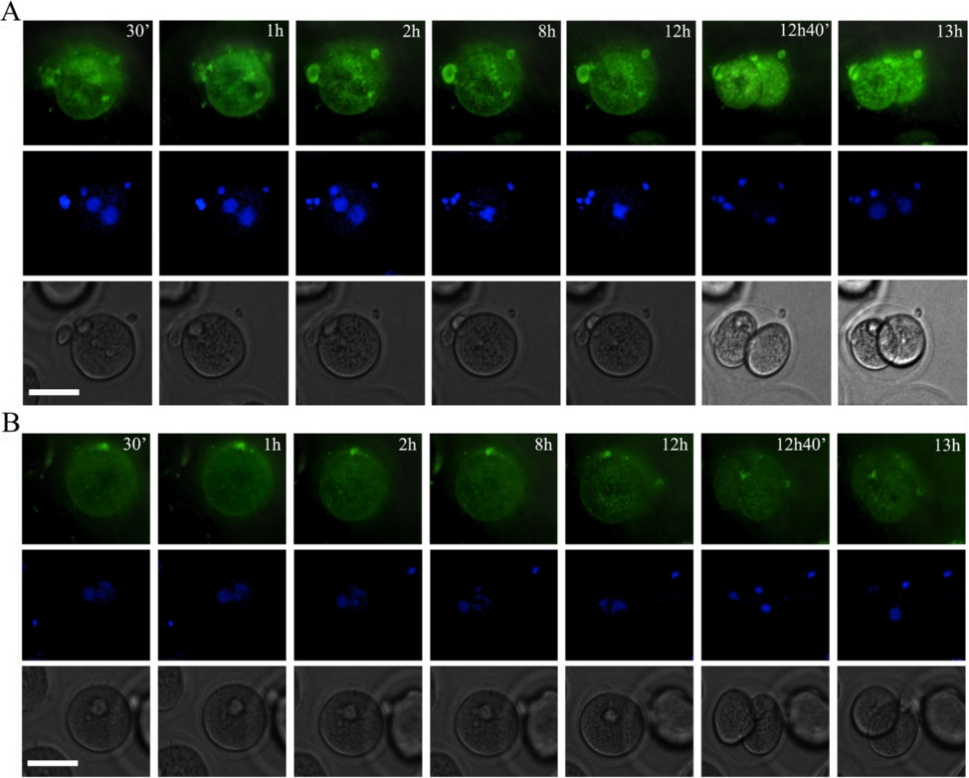
**ER redistribution was disrupted during development of early embryos in diabetic mice.** Time-lapse phase contrast microscopy (bottom row), ER tracker stained ER (top row) and Hoechst-stained chromosomes (middle row) of developing embryos from control mice (**A**) or diabetic mice (**B**). Times are indicated in the upper right corner. The scale bars represent 10 μm.

ER redistribution was disrupted in oocytes from diabetic mice during *in vitro* maturation. Therefore, we next examined the ER distribution patterns in *in vivo* oocytes at the GV stage, and at 8 h, and 14 h of hCG administration (Figure 1). As reported above, in the majority of control GV oocytes, a network of small ER accumulations throughout the cytoplasm was observed (75%, n = 40; Figure 3Ai, and Table 1), which we called the homogeneous distribution pattern. Following GVBD, ER displayed a perinuclear distribution pattern, characterized by the distinctive ring of fluorescence around the nucleus in prometaphase oocytes (77.1%, n = 35, Figure 3Bi and Table 2). Examination of MII oocytes at 14 hours of hCG revealed that the ER extended throughout the cytoplasm in a reticular organization pattern. There was no apparent labeling in the area assumed to be the meiotic spindle (78.9%, n = 38, Figure 5Ai and Table 3). In the cortex, there were accumulations of ER similar to those described previously [25]. Cortical cluster distribution of ER was only evident in MII oocytes (81.6%, n = 38, Figure 3Ai and Table 4).

**Table 1:** Comparison of ER distribution patterns in control or diabetic oocytes at the GV stage

**Group**	**No. oocytes examined**	**Percentage with I**	**Percentage with II**	**Percentage with III**
Control oocytes	40 (from 3 mice)	30 (75%)^a^	4 (10%) ^a^	6 (15%) ^a^
Diabetic oocytes	30 (from 3 mice)	5 (16.7%)^b^	14 (46.7%) ^b^	11 (36.7%) ^b^

* Experiments were repeated at least three times.

^a,b^ Values with different superscript letters are significantly different (P <0.05; Fisher exact test).

(I): homogeneous distribution pattern, (II): ER clouds, (III): cluster distribution patterns.

**Table 2:** Comparison of ER distribution patterns in control or diabetic oocytes at Pro-MII stage

**Group**	**No. oocytes examined**	**Percentage with I**	**Percentage with II**	**Percentage with III**
Control oocytes	35 (from 3 mice)	27 (77.1%) ^a^	5 (14.3%) ^a^	4 (11.4%) ^a^
Diabetic oocytes	30 (from 3 mice)	5 (16.7%) ^b^	18 (60.0%) ^b^	7 (23.3%) ^b^

* Experiments were repeated at least three times.

^a,b^ Values with different superscript letters are significantly different (P <0.05; Fisher exact test).

(I): perinuclear distribution pattern, (II): homogeneous distribution, (III): clustering distribution.

**Table 3:** Comparison of ER distribution patterns in equatorial section of control or diabetic oocytes at MII stage

**Group**	**No. oocytes examined**	**Percentage with left I**	**Percentage with left II**	**Percentage with left III**
Control oocytes	38 (from 3 mice)	30 (78.9%) ^a^	5 (13.2%) ^a^	3 (7.9%) ^a^
Diabetic oocytes	32 (from 3 mice)	6 (18.8%) ^b^	12 (37.5%) ^b^	14 (43.8%) ^b^

* Experiments were repeated at least two times.

^a,b^ Values with different superscript letters are significantly different (P <0.05; Fisher exact test).

(left I): no labeling in the area assumed to be the meiotic spindle poles, (left II): perinuclear distribution pattern, (left III): small areas of ER fluorescence within the deeper cytoplasm.

**Table 4:** Comparison of ER distribution patterns in cortical section of control or diabetic oocytes at MII stage

**Group**	**No. oocytes examined**	**Percentage with middle I**	**Percentage with middle II**
Control oocytes	38 (from 3 mice)	31 (81.6%) ^a^	7 (18.4%) ^a^
Diabetic oocytes	32 (from 3 mice)	7 (21.9%) ^b^	25 (78.1%) ^b^

* Experiments were repeated at least two times.

^a,b^ Values with different superscript letters are significantly different (P <0.05; Fisher exact test).

(middle I): cortical cluster distribution pattern, (middle II): without cortical clusters.

The distribution pattern of ER during *in vivo* meiotic maturation was disrupted in oocytes from diabetic mice. As shown in Figure 3 and Table 1, at the GV stage, the major ER redistribution defect related to the ER clouds (46.7%, n = 30) and cluster distribution pattern (36.7%, n = 30) . At the Pro-MI stage (Figure 3 and Table 2), a high frequency of homogeneous (60.0%, n = 30) and cluster distribution (23.3%, n = 30) of ER was observed in oocytes from diabetic mice. Concomitant with this finding, the percentage of ER distribution defects was also significantly increased in MII oocytes from diabetic mice, characterized by perinuclear distribution at the equatorial planes (37.5%, n = 32, Figure 4A and Table 3) or by small areas of ER fluorescence deeper within the cytoplasm (43.8%, n = 32; Figure 4A and B) and without cortical clusters at the cortical planes (78.1%, n = 32, Figure 4A and Table 4). Morphological parameters have been widely recognized as indicators of oocyte quality. We found that 19% of MII oocytes (n = 39) from diabetic mice displayed abnormal morphological characteristics (Figure 4A), including 1) enlarged perivitelline space, 2) giant polar bodies, and 3) fragmented cytoplasm, that was significantly higher than in controls (5.6%, n = 45; Figure 4A and B). Because oocytes with morphological abnormalities degenerate at a high frequency, only those oocytes with a normal appearance were selected for further analysis. Taken together, the above results suggest that maternal diabetes leads to inadequate redistribution of ER during oocyte maturation *in vivo* and adversely affects oocyte quality.

### ER redistribution was disrupted during early embryo development in diabetic mice

To determine whether maternal diabetes affects the ER distribution changes after fertilization, time-lapse imaging was performed in zygotes from control and diabetic mice. As shown in Figure 5A and supplemental video 4A (a representative zygote of a total of 20 pooled from 2 control mice), at various developmental stages of control fertilized oocytes, the spindles were located centrally and the pronuclear membranes were labeled with ER tracker. Following zygote division into two cells (Figure 5A and See Additional file 4: Supplemental Video S4), we observed larger areas of fluorescence deeper within the cytoplasm. The ER displayed a homogeneous distribution pattern throughout the entire ooplasm during development of embryos from diabetic mice (Figure 5B and See Additional file 5: Supplemental Video S5, a representative zygote of a total of 15 pooled from 2 diabetic mice).

**Figure 6:**
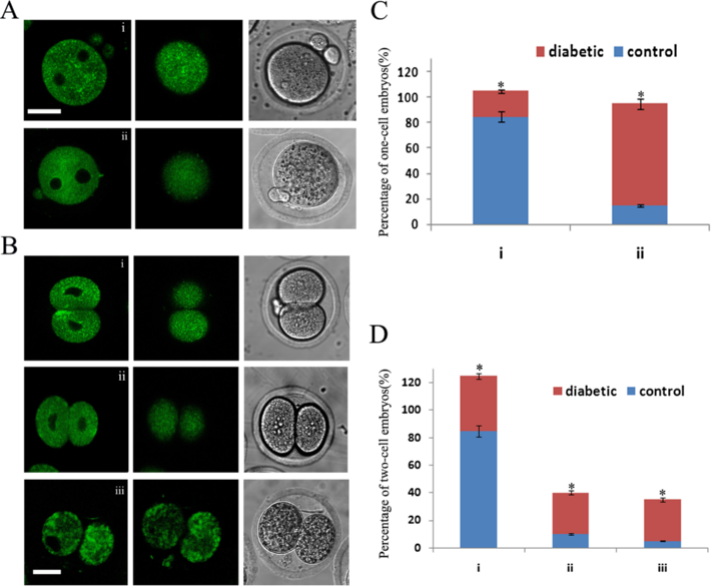
**Diabetes adversely affects ER redistribution during early embryo development.** Fertilized one-cell and two-cell stage embryos collected from control and diabetic mice were labeled with ER Tracker Green to visualize ER localization. ER distribution patterns were evaluated using fluorescence microscopy. **A**, in one-cell stage embryos, two distinct patterns of ER distribution were detected: perinuclear distribution, and larger areas of fluorescence deeper within the cytoplasm (i), homogeneous distribution pattern (ii); **B**, in two-cell oocytes, three different distribution patterns of ER were identified: perinuclear distribution pattern (i), homogeneous distribution (ii), and large aggregates of ER (iii). **C** and **D**, quantification of one-cell and two-cell stage embryos from each ER pattern of control and diabetic mice (n =30 for each group). Data are presented as mean ± SEM from three independent experiments. *, P < 0.05 vs. controls. Bar = 20 μm.

To examine more precisely the different ER distribution events in embryos from control and diabetic mice we compared the ER staining patterns in the equatorial xplane and cortical clusters of ER with confocal laser scanning microscopy. We found that the majority of PN zygotes from control mice displayed a striking ER localization around the pronuclei in the equatorial section which was significantly increased when compared to zygotes from diabetic animals (Figure 6Ai and C). In addition, the proportion of the larger areas of fluorescence deeper within the cytoplasm was significantly increased in zygotes from control mice compared to those from diabetic mice (85 ± 14%, n = 30 vs. 20 ± 4%, n = 30, Figure 6Ai and C). In contrast, more than 80% one-cell (n = 21) and 40% two-cell (n = 13) stage embryos from diabetic mice exhibited a higher frequency of the homogeneous ER distribution pattern. Similar ER distribution was displayed in 2-cell stage embryos from control or diabetic mice (Figure 6Bi, B ii and D, n = 30). Furthermore, we found that 30 ± 7% of two-cell embryos from diabetic mice showed very large aggregates of ER throughout the cytoplasm. Most of them were unable to develop and completely deteriorated within a short time. These observations suggest that the ER redistribution is disrupted during early embryo development in diabetic mice and may play a role in reproductive failure and congenital birth defects.

## Discussion

The ER is a dynamic structure, capable of changing its cellular organization and distribution patterns remarkably as shown at fertilization of starfish and sea urchin eggs. Here using time-lapse live imaging confocal microscopy, we showed that mouse oocytes undergo a dramatic reorganization of ER during meiotic maturation *in vitro* and *in vivo.* GV-stage oocytes contained a fine ER network throughout the interior cytoplasm and cortex. Following GVBD, ER surrounded the spindle during its migration to the oocyte cortex. MII oocytes contained striking ER accumulations at the cortex, with no apparent polarity in relation to the meiotic spindle, similar to those described previously [19,24,25]. On the other hand, we first revealed that maternal diabetes is associated with inadequate translocation of ER during oocyte maturation and a high proportion of oocytes from diabetic mice showed morphological abnormalities. Morphological parameters have been widely recognized as an indicator of oocyte quality. In our study, we showed that oocytes with morphological abnormalities degenerated at a high frequency, and only those oocytes with a normal appearance were selected for further analysis. We clearly observed a homogeneous distribution of ER throughout the entire ooplasm during the meiotic maturation process in oocytes from diabetic mice. ER distribution is an indicator for cytoplasmic maturation [26]. Studies have shown that spatial remodeling of endoplasmic reticulum render the oocyte capable of supporting development [24]. The ER is a vast membranous network responsible for protein synthesis and assembly, maturation, and along with the Golgi apparatus, transportation and release of correctly folded proteins. It is also a critical site for Ca^2^+ homeostasis [10,27]. Ca^2+^-ATPases, Ca^2+^ storage proteins, and specific Ca^2+^ release channels have been localized to the ER, which permits this organelle to perform a crucial role in the regulation of intracellular Ca^2+^[28,29]. Moreover, the ER contains InsP3 receptors and, in some cases, ryanodine receptors, both of which mediate Ca^2+^ release from the ER [30]. A specialized ER organization in MII mouse oocytes are the cortical ER clusters which act as pacemaker sites for the generation of Ca^2+^ oscillations at fertilization [31,32]. The vegetal cortex is more sensitive to InsP3 and Ca^2+^-releasing sperm extracts [21] and acts as the Ca^2+^ wave pacemaker at fertilization [22,31] and even after fertilization near the spindle [33]. The localization of InsP3Rs to the ER clusters suggests an important role in regulating the initiation of Ca^2+^ release [20,33]. Clustering of InsP3Rs in ER increases the sensitivity of Ca^2+^ release such that coherent signals can be generated in response to very low levels of stimuli that otherwise would not elicit a response [34]. This may be particularly pertinent to fertilization of mammalian eggs where low InsP3 concentrations have been proposed [35] and where the signaling pathway involves the introduction of a phospholipase C from a very small cell (the sperm) into a very large cell (the egg) [35]. These observations demonstrate that the cortical ER clusters play an important role in the initiation and spatial organization of Ca^2+^ signaling at fertilization. Thus, we can conclude that inadequate redistribution of ER may be one of the important factors contributing to the maturation delay and spindle/chromosome disorganization observed in diabetic oocytes. However, previous studies revealed that ovulated oocytes from diabetic mice displayed an alteration in mitochondrial ultrastructure, and quantitative analysis of mitochondrial DNA copy number demonstrated an increase [10]. Therefore, the defects in diabetic oocytes might be the interaction between the ER and mitochondria. Taken together, the above results suggest that maternal diabetes leads to inadequate redistribution of ER during oocyte maturation in vitro and in vivo.

As a central regulator of protein quality control, folding, trafficking, and targeting, the ability of the ER to adapt its capacity to manage synthetic, metabolic, and other adverse conditions is of paramount importance for the cell [29]. In the present study we found spindle-associated ER as well as larger areas of ER-fluorescence deeper within the cytoplasm in mouse early embryos. ER displayed a homogeneous distribution pattern throughout the entire ooplasm during development of embryos from diabetic mice. We conclude that the impaired organization of the ER could account for the reduced developmental potential observed in early embryos from diabetic mice.

In addition, very large ER aggregations were seen in GV oocytes or in two-cell embryos from diabetic mice. First, we found that GV oocytes from diabetic mice displayed a significantly higher percentage of aggregated ER distribution areas near the nucleus when compared with controls. These oocytes were not able to resume meiotic maturation and completely deteriorated within a short time. Second, we found that two-cell embryos from diabetic mice showed a higher percentage of very large aggregated ER throughout the cytoplasm when compared to controls. Importantly, most of them were unable to develop further. The ER elicits an elaborate adaptive response known as the unfolded protein response (UPR). Much of the systemic physiology related to its dysfunctions has been viewed in the context of its luminal adaptation to protein processing and folding. In eukaryotic cells, monitoring of the ER lumen and the canonical branches of the UPR are mediated by three ER membrane-associated proteins. In a well-functioning and “stress-free” ER, these transmembrane proteins are bound by a chaperone in their intraluminal domains and rendered inactive [36,37]. There are three mechanisms to mitigate ER stress which includes reducing protein synthesis, facilitating protein degradation, and increasing production of chaperones that help proteins in the ER lumen to fold. These mechanisms are implicated in resolving stress; if they fail the cell becomes functionally compromised and may undergo apoptosis. Together with our findings, we propose that the very large aggregated ER in oocytes or early embryos may contribute to ER stress and UPR, and hence commit to cell death which needs further detailed investigation.

## Conclusions

In summary, our results show that maternal diabetes disrupts the ability of the ER to reorganize during oocyte maturation and early embryo development, and this could contribute to the reproductive problems experienced by type I diabetic mice as well as women. Our findings may have clinical implications for the assessment of oocyte quality from diabetic women. For example, polarization microscopy [38] can be used to directly recognize those diabetic oocytes with abnormal ER distribution; therefore it may be possible to screen diabetic oocytes to choose oocytes or embryos with optimal quality, finally, targeting drugs to improve ER function in oocytes may have therapeutic potential in treating reproductive failure of diabetic women.

## Competing interests

The authors declare that they have no competing interests.

## Authors’ contributions

Conceived and designed the experiments: CHZ QYS HS. Performed the experiments: CHZ, WPQ, STQ, ZJG, LJM, XLZ, XH and JPL. Analyzed the data: CHZ WPQ QYS. Contributed reagents/materials/analysis tools: YCO YH. Wrote the paper: CHZ QYS HS. All authors read and approved the final manuscript.

## Supplementary Material

Additional file 1 **Supplemental Video S1.** Mouse GV oocytes were incubated in KSOM/AA medium containing 100-500 mM ER tracker and 10 nM Hoechst 33342 dye in KSOM/AA medium and allowed to undergo meiotic maturation. The time-lapse images were acquired every 30 min for 10–12 hr (Additional file 1: Video S1). Click here for file

Additional file 2 **Supplemental Video S2.** Similar to Video 1A, the dynamic changes of ER in oocytes from diabetic mice are shown in Additional file 2: Video S2. Click here for file

Additional file 3 **Supplemental Video S3.** Similar to Video 1A, the dynamic changes of ER in oocytes from diabetic mice are shown in Additional file 3: Video S3. Click here for file

Additional file 4 **Supplemental Video S4.** Zygotes collected from the control were labeled with ER Tracker Green to visualize the dynamic localization of ER during early embryo development. The time-lapse images were acquired every 30 min for 13–15 hr. Click here for file

Additional file 5 **Supplemental Video S5.** Similar to Video 4, zygotes collected from the diabetic mice were labeled with ER Tracker Green to visualize the dynamic localization of ER during early embryo development. Click here for file
